# What Is the Role of Palmitoylethanolamide Co-Ultramicronized with Luteolin on the Symptomatology Reported by Patients Suffering from Long COVID? A Retrospective Analysis Performed by a Group of General Practitioners in a Real-Life Setting

**DOI:** 10.3390/nu15173701

**Published:** 2023-08-24

**Authors:** Maurizio Pirro, Luana Ferri, Licia Piccioni, Anna Maria Bellucci, Federica Bartolucci, Arianna Russo, Andrea Piga, Paola Lucia Ciaramaglia, Marco Lucangeli, Anna Maria Russo, Salvatore Cuzzocrea, Maurizio Evangelista

**Affiliations:** 1 Azienda Sanitaria Locale (ASL), Sistema Sanitario Regionale, 00012 Rome, Italy; 2 Institute of Anaesthesiology and Reanimation, Catholic University of Sacred Heart, 00168 Rome, Italy; 3 Department of Chemical, Biological, Pharmaceutical and Environmental Sciences, University of Messina, 98166 Messina, Italy; 4 Department of Clinical and Experimental Medicine, University of Messina, 98166 Messina, Italy

**Keywords:** long COVID, neuroinflammation, PEALUT, real-life setting, pain, brain fog, anosmia, dysgeusia, fatigue

## Abstract

Long COVID is a recognized post-viral syndrome characterized by neurological, somatic and neuropsychiatric symptoms that might last for long time after SARS-CoV-2 infection. An ever-growing number of patients come to the observation of General Practitioners complaining of mild or moderate symptoms after the resolution of the acute infection. Nine General Practitioners from the Rome area (Italy) performed a retrospective analysis in order to evaluate the role of the supplementation with Palmitoylethanolamide co-ultramicronized with Luteolin (PEALUT) on neurologic and clinical symptoms reported by their patients after COVID-19 resolution. Supplementation with PEALUT helped to improve all patient-reported symptoms, especially pain, anxiety and depression, fatigue, brain fog, anosmia and dysgeusia, leading to an overall improvement in patients’ health status. To our knowledge these are the first data presented on Long COVID patients collected in a territorial setting. Despite their preliminary nature, these results highlight the pathogenetic role of “non-resolving” neuroinflammation in Long COVID development and consequently the importance of its control in the resolution of the pathology and put the focus on the General Practitioner as the primary figure for early detection and management of Long COVID syndrome in a real-life setting. Future randomized, controlled, perspective clinical trials are needed to confirm this preliminary observation.

## 1. Introduction

Long COVID is a recognized post-viral syndrome characterized by neurological, somatic and neuropsychiatric symptoms, that might last for long time after SARS-CoV-2 infection and can significantly interfere with daily living activities [1,2].

The literature generally reports about 10% of infected people who develop Long COVID, even if in some works the prevalence rises between about 20% and 45% [3,4,5].

More and more often General Practitioners are dealing with patients who had contracted SARS-CoV-2 and after infection resolution still complain of various symptoms, from mild to moderate. Its main symptomatology includes painful syndromes onset (neuropathic, musculoskeletal, nociplastic pain) or the worsening of chronic existing one, a sense of fatigue, weakness, anosmia and dysgeusia persistence, depression, anxiety and cognitive disfunctions like brain fog and memory impairment [6,7,8,9,10].

Long COVID condition is the consequence of an inflammatory process characterized by an excessive leucocyte infiltration and an impressive release of cytokines, chemokines and proteases, promoters of inflammation [11]. A lot of evidence shows that, together with macrophages and neutrophiles, mast cells (MC) play a relevant role in the onset of a peripheral neuroinflammatory process, inducing secretion and synthesis “ex-novo” of pro-inflammatory mediators [12,13,14]. The systemic inflammatory storm also causes a significant alteration of the blood–brain barrier (BBB) inducing hyperactivation of microglia, the immune cells resident in the central nervous system (CNS) [15,16,17,18,19]. The consequence is the triggering of neuroinflammation at the central level, with implications that can be very serious especially in elderly subjects in which, due to the neuroinflammatory state that accompanies aging (inflammaging) and the concomitant neuroinflammatory discrepancy given by SARS-CoV-2 infection, it becomes even more fundamental to act in prevention to avoid exacerbations of ongoing cognitive decline [20,21,22].

For all these reasons, it becomes crucial to find new approaches to control as early as possible the neuroinflammatory process acting on the immune system regulation. From this perspective, a putative intervention might be represented by Palmitoylethanolamide (PEA). PEA is an endogenous molecule belonging to the *N*-acylethanolamine family, synthetized “on demand” in response to stress factors to restore the tissue homeostasis, exerting its protective action thanks its ability to modulate mast cell hyperactivation through the so-called ALIA “Autacoid Local Injury Antagonism” mechanism [23]. At the molecular level, PEA interacts directly with PPAR-α (Peroxisome Proliferator-Activated Receptor alpha) and GPR55 (G Protein-Coupled Receptor 55) receptors and indirectly, through an “entourage effect”, on CB1, CB2 (Cannabinoid receptor type 1 and 2) and TRPV1 (Transient Receptor Potential Vanilloid 1) [24,25,26,27,28]. In addition, the above profile of palmitoylethanolamide and luteolin shows that both compounds are potent antioxidants and neuroprotectors [29].

A very large number of publications in the preclinical field have documented its efficacy in numerous pathologies which, although with different aetiologies and topographies, are united by the pathogenetic mechanism of non-resolving neuroinflammation.

Due to its lipophilic nature and large particle size in the native state, PEA presents limitations in terms of solubility and bioavailability when given orally. For this reason, PEA for oral administration is micronized (mPEA) and ultramicronized (umPEA), through a process which, by reducing its particle size, improves its absorption and distribution, increasing its biological efficacy [30,31]. Many clinical and preclinical studies conducted on chronic pain and neurodegenerative syndromes characterized by a shared neuroinflammatory pathogenic mechanism (neuropathic pain, nociplastic pain, chronic pain, fibromyalgia, Alzheimer’s and Parkinson’s disease, multiple sclerosis and sleep–wake rhythm disturbances) have demonstrated the efficacy of m/umPEA in improving the overall clinical picture [32,33,34,35,36,37,38,39,40]. UmPEA efficacy has been proved also in COVID-19 paucisymptomatic patients, where it reduced inflammatory and oxidative stress markers [41].

Furthermore, it has been demonstrated that the co-ultramicronization between umPEA and specific polyphenols like polydatin or luteolin originates microcomposites with higher neuroprotective, anti-inflammatory and antioxidant proprieties [42,43,44]. Co-ultramicronization of PEA with luteolin (PEALUT) proved to be a high-potential therapeutic instrument for the treatment of several diseases characterized by the presence of neuroinflammation [45,46,47] and similarly also in COVID-19 sequelae, where following its oral treatment it was achieved an improvement in anosmia and mental clouding [48,49,50] and also the restoration of GABAB-ergic activity and cortical plasticity in Long COVID patients [51].

Consistently with what has already been observed in other clinical conditions, neuro-immuno-inflammation is not only underlying the COVID-19 disease, but is also the main cause of its sequelae that affect and compromise the respiratory apparatus and also the nervous and gastrointestinal systems, inducing new clinical pictures or aggravating pre-existing ones. Based on this evidence, a group of General Practitioners decided to retrospectively analyse data about their Long COVID patients, to verify the role of PEALUT supplementation in syndrome resolution.

## 2. Materials and Methods

Nine General Practitioners from the Rome area (Italy) retrospectively analysed the clinical chart of their Long COVID patients that during 2022 were treated with the Food for Special Medical Purpose (FSMP) PEALUT (Glialia^®^, Epitech Group SpA, Saccolongo, Italy) in order to control symptomatology arisen after SARS-CoV-2 infection.

PEALUT was prescribed according to its indications for use and the existing literature data at the dosage of 700 mg + 70 mg, 2 sachets a day for 90 days, as an add-on to concomitant therapy performed by patients (antihypertensives, anticoagulants, antithyroids drugs, etc.) [48,49,50].

Patients ≥ 18 years of age, of both genders, who had experienced COVID-19 in the previous 2–4 months, with at least two neurological or physical symptoms (pain, depression, anxiety, fatigue, brain fog, anosmia, or other clinical symptoms) that occurred after SARS-CoV-2 infection, were considered for this analysis. No patients with serious medical conditions, neuropsychiatric pathology or pain syndromes that could have interfered with the interpretation of the results, were included. None of the patients considered had been hospitalized during the acute phase of COVID-19 and, at the time of treatment, none were taking any specific therapy for the reported symptoms. Considered patients did not change ongoing therapies for their concomitant pathologies (mild hypertension, cardiovascular pathologies and/or thyroiditis) nor did they start new treatments during the entire observation period (pharmacological, with other supplements or rehabilitative, neither physical nor psychological). No pregnant or breastfeeding women were considered.

The symptoms reported by the patients and detected at the time of the first visit (T0) were present and persisted for at least eight weeks after the swab was negative. None of the symptoms were present before the SARS-CoV-2 infection and COVID-19; in the period preceding the first visit (T0), and none of the patients had received therapy, either specific or general. 

During the observation period, patients were evaluated through a battery of validated self-administered tool in order to evaluate their overall improvement. All examinations were performed prior to starting treatment with PEALUT (T0), after 60 and 90 days (T60 e T90) according to the clinical practice in force in the outpatients clinic involved.

Evaluations included:

1. Intensity and type of painful symptoms by means of the Pain Detect Questionnaire (PD-Q), a validated tool for the screening of neuropathic pain in various clinical conditions, and subsequently also for the monitoring of patients clinical course [52]. PD-Q is a self-reported questionnaire divided into four sections: pain intensity, pain course pattern, presence/absence of radiating pain and sensory symptoms evaluation. Pain intensity is evaluated by three questions about pain at the moment “current pain”, the strongest “last month pain” and the mean “last month pain”. Each one is assigned a score by an 0–10 NRS (Numeric Rating Scale) where 0 represents “no pain” and 10 a “maximum intensity pain”. Pain course pattern is evaluated by four graphs that represent the four possible options (only one answer possible): persistent pain with slight fluctuations (0 points), persistent pain with pain attacks (−1 point), pain attacks without pain between them (+1 point) and pain attacks with pain between them (+1 point). Radiating pain evaluation is a unique yes/no question about the presence (+2 points) or absence (0 points) of pain radiations; a body chart drawing allows a patient to indicate the direction in which the pain radiates. Sensory symptoms evaluation consists of seven questions about seven items: burning, tingling or prickling sensation, dynamic mechanical allodynia, electric shock, thermal hyperalgesia, numbness and static (pressure) allodynia. Each of them is evaluated by a 0–5 scale where 0 indicates “never noticed” and 5 “very strong”. The score of this section varies from 0 to 35 and represents the PD-Q Total Score. Adding to this Total Score the points obtained from the previous two sections: pain course pattern (−1, 0 or +1) and radiating pain (0 or +2), it is possible to calculate the PD-Q Final Score, used for discriminate the type of pain from “nociceptive” (0–12), “unclear” (13–18) and “neuropathic” (19–38) [53].
2. Depression and anxiety symptoms by means of the Hamilton Rating Scale for Depression (HAM-D) and anxiety (HAM-A). HAM-D is a questionnaire which assesses the presence and severity of depressive symptoms through the evaluation of 21 items. A score <7 indicates absence of depression; 8–17 correspond to mild depression, 18–24 to moderate and >25 severe [54]. HAM-A is a questionnaire consisting of 14 symptom-defined items, scored on a scale of 0 (not present) to 4 (severe), with a total score range of 0–56 where <17 indicates mild severity, 18–24 mild to moderate and 25–30 moderate to severe [55].
3. Long COVID symptomatology by evaluating the persistence or the new onset of clinical signs/symptoms recognized as sequalae of COVID-19 pathology, like brain fog, difficulty multitasking, fatigue, irritability, inability to find the right word, memory loss and weakness [56], in addition to the recognized olfactory and gustatory alteration anosmia and dysgeusia [57].
4. Patients subjective improvement at the end of the study by the Patient Global Impression of Change (PGIC). PGIC is a validated tool easy to apply in daily clinical practice for the non-research clinician to quantify and monitor patient progress and treatment response over time [58]. PGIC evaluates the variation of the health status perceived by each patient through seven possible options ranging from “extremely worse” to “extremely improved” [59].

Statistical analysis was carried out by GLMM (General Linear Mixed Model) for the evaluation of treatment efficacy over time. A post hoc analysis using a Tukey–Kramer multiple comparisons test was performed to evaluate treatment efficacy at each time point compared to the previous one (T60 vs. T0 and T90 vs. T60). Values are expressed as mean ± standard error (S.E.) or standard deviation (S.D). A *p*-value of less than 0.05 was considered statistically significant.

## 3. Results

Forty-nine outpatients (18 males and 31 females) with an average age ± S.D. of 53.4 ± 12.0 years were considered in this retrospective analysis. Patients baseline characteristics are reported in Table 1 and Table 2. No side-effects were reported during treatment, nor any pharmacological interactions with concomitant medications.

### 3.1. Pain Evaluation by Pain Detect Questionnaire (PD-Q)

In this study, the results of the first three sections of the PD-Q questionnaire are reported.

#### 3.1.1. Pain Intensity

Pain intensity, self-evaluated by patients at T0 through the three different PD-Q NRS (“current pain”, “strongest last month pain” and “average last month pain”) showed the presence of pain of a mild-to-moderate intensity, progressively attenuated during the 90 days of observation, reaching values no longer clinically relevant by the end of treatment. 

**Figure 1:**
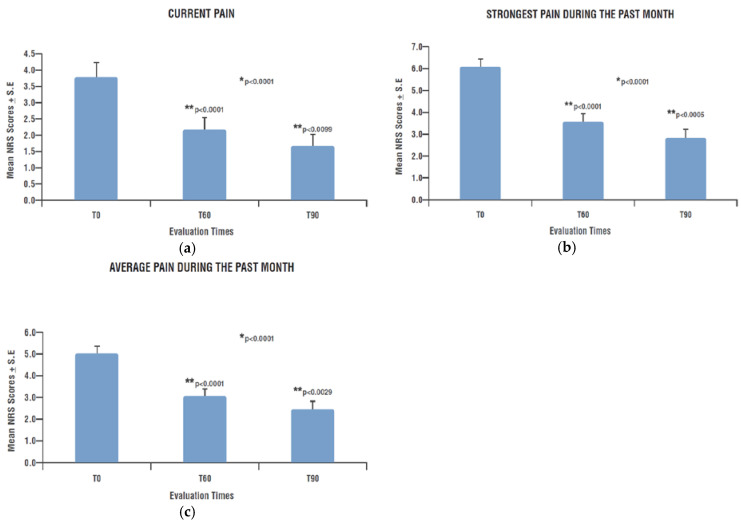
Pain intensity over time (PD-Q NRS scores). (**a**) Current pain intensity at each time-point; (**b**) strongest pain intensity during the previous month at each time-point; (**c**) average pain intensity during the previous month at each time-point. * Significant change over time (GLMM); ** Significant change compared to previous assessment (post hoc analysis). PD-Q: Pain Detect Questionnaire; NRS: Numerical Rating Scale; T0: examination prior to start treatment with PEALUT; T60: examination after 60 days of treatment with PEALUT; T90: examination after 90 days of treatment with PEALUT.

In particular, the mean NRS scores decreased from 3.8 ± 0.44 (T0) to 2.2 ± 0.36 (T60) and to 1.7 ± 0.36 (T90) for the “current pain” (T60 vs. T0 *p* < 0.0001; T90 vs. T60 *p* < 0.0099) (Figure 1a); from 6.1 ± 0.34 (T0) to 3.6 ± 0.37 (T60) and to 2.8 ± 0.38 (T90) for the “strongest last month pain” (T60 vs. T0 *p* < 0.0001; T90 vs. T60 *p* < 0.0005) (Figure 1b) and from 5.0 ± 0.35 (T0) to 3.1 ± 0.33 (T60) and to 2.5 ± 0.36 (T90) for the “average last month pain” (T60 vs. T0 *p* < 0.0001; T90 vs. T60 *p* < 0.0029) (Figure 1c). 

The decrease in pain intensity was statistically significant over time for all the three types of pain (*p* < 0.0001 for each one). Furthermore, as shown by the *p*-values reported above, the post hoc analysis highlighted that at each time point, pain intensity decreased in a statistically significative manner in comparison to the previous one (Figure 1a–c).

#### 3.1.2. Pain Course Pattern and Radiating Pain Evaluation

The pain course pattern evaluation showed that at T0 only 8.2% of patients reported the absence of pain, while at T90 the number of patients who did not experience pain increased to 33.3%. “Persistent pain with pain attacks” and “pain attacks with pain between them” patterns showed an improvement over time: the percentage of patients who experienced these types of pain decreased from 16.3% at baseline to 6.5% at T60 and to 4.8% at T90 and from 18.4% at T0, to 10.9% at T60 and to 9.5% at T90, respectively. “Persistent pain with slight fluctuations” was presented by 22.4% of patients at T0, 28.3% at T60 and 21.4% at T90. Similar results were reported for “pain attacks without pain between them”: 34.7% patients experienced this type of pain at T0, 23.9% at T60 and 31.0% at the end of the observation period (Table 3).

Among patients who answered the question about radiating pain at baseline, 36.2% described their pain as radiating. Pain irradiation was reported by 26.1% patients at T60 and 38.1% at T90.

### 3.2. Depression and Anxiety Symptoms by Hamilton Rating Scale for Depression (HAM-D) and Anxiety (HAM-A)

**Figure 2:**
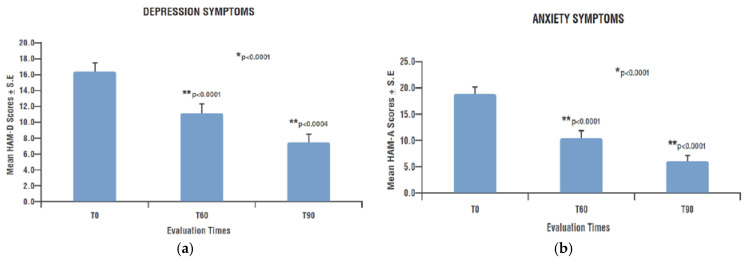
Depression and anxiety symptoms over time. (**a**) HAM-D score; (**b**) HAM-A score. * Significant change over time (GLMM); ** Significant change compared to previous assessment (post hoc analysis). HAM-D: Hamilton Depression Questionnaire; HAM-A: Hamilton Anxiety Questionnaire; T0: examination prior to start treatment with PEALUT; T60: examination after 60 days of treatment with PEALUT; T90: examination after 90 days of treatment with PEALUT.

Depressive and anxious symptomatology evaluated by the HAM-D and HAM-A questionnaires became progressively and significantly weaker during the observation period and at each time point compared to the previous one. HAM-D score significantly decreased from a mean value of 16.4 ± 1.04 (T0) to 11.2 ± 1.16 (T60) and to 7.5 ± 1.03 (T90) (*p* < 0.0001) (T60 vs. T0 *p* < 0.0001; T90 vs. T60 *p* < 0.0004) (Figure 2a). HAM-A score significantly decreased over time from a mean value of 18.9 ± 1.27 (T0) to 10.6 ± 1.33 (T60) and to 6.1 ± 1.14 (T90) (*p* < 0.0001) (T60 vs. T0 *p* < 0.0001; T90 vs. T60 *p* < 0.0001) (Figure 2b).

### 3.3. Long COVID Symptomatology

The presence and distribution of the assessed clinical symptoms such as anosmia, dysgeusia, brain fog, difficulty multitasking, fatigue, irritability, inability to find the right word, memory loss and weakness, significantly improved during the treatment period. 

**Figure 3:**
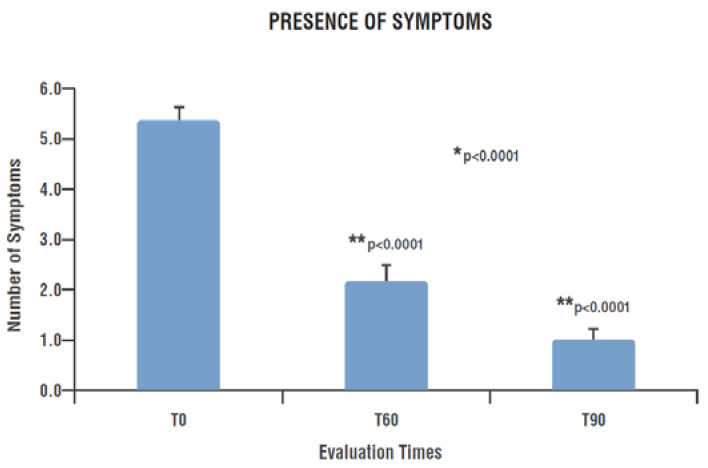
Number of symptoms presented by patients at each time-point. * Significant change over time (GLMM); ** Significant change compared to previous assessment (post hoc analysis). T0: examination prior to start treatment with PEALUT; T60: examination after 60 days of treatment with PEALUT; T90: examination after 90 days of treatment with PEALUT.

In particular, patients presented a mean of 5.4 ± 0.26 symptoms at T0, decreased to 2.2 ± 0.31 at T60 and to 1.0 ± 0.22 at the end of treatment (T90) (*p* < 0.0001). This reduction was statistically significant already after 60 days of treatment (T60), showing a further significant decrease at T90 (T60 vs. T0 *p* < 0.0001; T90 vs. T60 *p* < 0.0001) (Figure 3).

**Figure 4:**
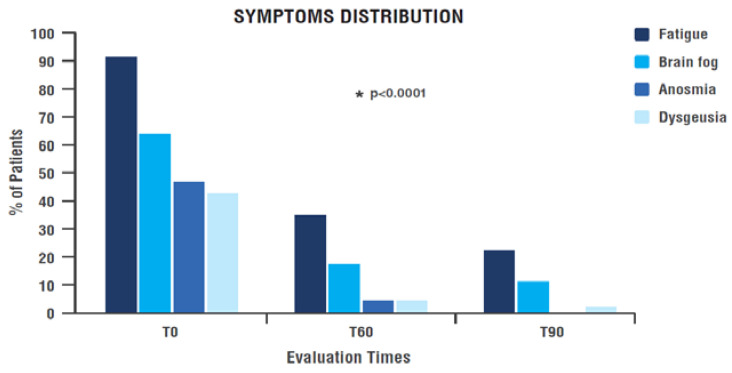
Symptom distribution among patients over time. * Significant change over time (GLMM). T0: examination prior to start treatment with PEALUT; T60: examination after 60 days of treatment with PEALUT; T90: examination after 90 days of treatment with PEALUT.

The percentage of patients complaining of each evaluated symptom decreased a lot from the beginning (T0) to the end of treatment (T90) in a significant manner over time for every item considered (Figure 4 and Table 4). In particular:

- Anosmia and dysgeusia reported by 46.8% and 42.6% of patients, respectively at the time of enrolment, were almost absent after 90 days of treatment with PEALUT: no patients reported anosmia and only one patient still had dysgeusia (*p* < 0.0001) (Figure 4 and Table 4).
- Fatigue was reported by 91.5% of patients at T0, showing a significant improvement over time (*p* < 0.0001). During treatment period the percentage of patients reporting fatigue dropped to 34.8% at T60 and to only 22.2% at T90 (Figure 4 and Table 4).
- Brain fog distribution among patients achieved a marked and significant improvement during treatment (*p* < 0.0001): the percentage of patients reporting this symptom dropped from 63.8% at T0 to 17.4% at T60 and 11.1% at T90 (Figure 4 and Table 4).

Regarding all the other symptoms considered, at the end of the observation period, out of 45 patients, only 5 (11.1%) reported difficulty multitasking, memory loss and inability to find the right word, compared to 28 (59.6%), 24 (51.1%) and 20 (42.6%) at T0; only 7 patients (15.6%) showed weakness and irritability, versus 35 (74.5%) and 30 (63.8%) at basal. Distribution of each considered symptoms among patients showed a statistically significant decrease over time, as reported in Table 4.

### 3.4. Patients Subjective Improvement (PGIC Scale)

**Figure 5:**
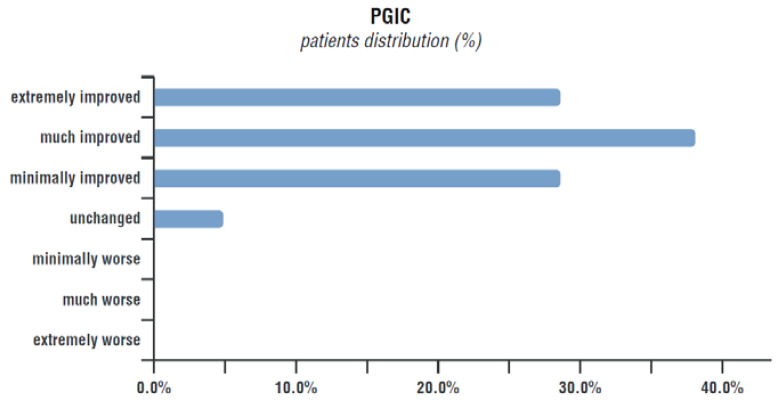
Change in the health status perceived by patients according to the PGIC scale at the end of treatment.

According to PGIC results, at the end of the therapy 95.3% of patient felt improved; in particular, 32.4% of patients declared themselves to be “extremely improved”, 38.1% of patients reported they were “much improved”, 28.6% considered themselves as “minimally improved”. Only 4.8% of them declared they felt “unchanged” and no patients complained of being worse (Figure 5).

## 4. Discussion

Long COVID, according to WHO definition, is a clinical condition that occurs in individuals with a history of probable or confirmed SARS CoV-2 infection, usually 3 months from the onset of COVID-19 with symptoms and that last for at least 2 months and cannot be explained by an alternative diagnosis [3]. It manifests with symptoms of various intensity and duration affecting many systems and organs, and generates important repercussions not only in the medical but also psychological, economic and social field [19]. A recent review published on Nature reported that at least 65 million people worldwide have already experienced this pathology [4] and projections of the most authoritative scientific literature estimate that Long COVID will affect in the future more than 200 million people, mainly between 20 and 50 years old. In August 2022 Brookings Institution calculated that Long COVID was keeping the equivalent of two million to four million full-time workers out of the American labour force, resulting in about $170 billion of lost earnings per year [60]. Economist David Cutler drew similar conclusions, estimating Long COVID-related costs on the order of $100 billion per year for the U.S. health system [61]. Combining these data, it will result in a cost of more than $1 trillion over a five-year period, even before accounting for lost quality of life, increased disability costs and the burden on caregivers and the health care system [62].

Clinically, Long COVID comprehends the onset of various conditions, including cardiovascular, thrombotic, and cerebrovascular diseases, type 2 diabetes mellitus, myalgic encephalomyelitis/chronic fatigue syndrome (ME/CFS), and dysautonomia, particularly postural orthostatic tachycardia. Possible neuropathology includes coagulopathy-related blood vessel damage, endothelial dysfunction, and neuronal injury, with symptoms that can last for years and even life [4,63]. Furthermore, in addition to those more known and clinically defined, there are several minor dysfunctions documented, such as fatigue, anxious and depressive symptomatology, loss of smell and taste, and the persistence or worsening of musculoskeletal pain, that have a huge impact on people’s quality of life in all its global and daily aspects [1,2,4,5,6,49,50]. In addition to this, cognitive dysfunctions such as brain fog, difficulty concentrating, memory impairment, and problems with speech and language are the most common symptoms reported during Long COVID, occurring in around 70% of patients and second only to fatigue [64]. The responsible for the nonspecific/subjective and persistent symptoms exhibited by some patients, typically out of proportion compared to the degree of lung damage, might be the massive release of cytokines, interleukins and other pro-inflammatory molecules and the subsequent onset of a neuroinflammatory status that characterizes COVID-19 pathology [65].

Currently there are no effective validated treatments for Long COVID syndrome: mechanistic studies are generally in early stages and although research based on post-viral diseases such as ME/CFS has led to some speculation, many questions remain unsolved [4]. This made the scientific community question itself to find a strategy to manage this poorly defined pathology, already capable of generating such a serious bio-psycho-socio-economic systemic burden.

Among the new strategies that have emerged in the treatment of Long COVID, the use of umPEA in combination with luteolin stands out. PEALUT is a neuroprotective compound recently studied in patients with COVID-19 and Long COVID syndrome with excellent results [48,49,50,51]. In this perspective, a group of General Practitioners from Rome area (Italy) performed a retrospective analysis on their Long COVID patients, in order to evaluate PEALUT efficacy on complained symptomatology. According to PD-Q questionnaire results, patients came to the General Practitioner experiencing pain of a mild-to-moderate intensity occurring after SARS-CoV-2 infection and showed a statistically significant improvement over time after starting PEALUT treatment, reaching values no longer clinically relevant by the end of observation period. Both anosmia and dysgeusia, presented by more than 40% of patients at baseline, were almost absent at the end of the treatment. The same occurred for fatigue, one of the main sequelae of Long COVID, reported by 91.5% of patients at the basal state and in only 22.2% at the end of treatment. Similar results were obtained for weakness, brain fog, irritability and all evaluated symptoms, whose presence drastically decreased, becoming irrelevant at the end of observation period. Moreover, the presence of anxiety and depression of mild severity in the enrolled patients, highlighted by HAM-D and HAM-A score analysis, was significantly decreased after PEALUT supplementation. The resolution of analysed symptomatology was accompanied by a marked amelioration in the state of health perceived by patients, as demonstrated by the results obtained from the PGIC scale, with over 95% of patients considering their health improved upon completion of PEALUT treatment.

The current clinical findings strongly support the previous preclinical studies and emphasize the importance of work to be conducted on these compounds for the management of COVID-related complications.

To our knowledge, these are the first data on Long COVID patients not previously hospitalized, collected in a territorial setting. It is noteworthy that the doctor–patient relationship, characterized by a familiar approach (less delayed by logistical and bureaucratic disadvantages), allowed the early identification of Long COVID patients, which allowed us to counteract the neuroinflammatory process in its initial phase.

Our current healthcare system is actually fragmented, symptom- and specialty-focused and, therefore, not suitable for the management of Long COVID. The collection of such a myriad of symptoms requires a range of subordinated specialist care becoming increasingly inadequate due to the 5.6 million patients awaiting hospital treatment in the UK alone. The first “landing point” for many patients is usually their General Practitioner, from whom they need confidence, empathy and understanding and whose support, during recovery and rehabilitation from COVID-19, is even more essential [66]. This suggests that, at least in the prodromal phases, the General Practitioner could be the most appropriate figure in the territorial setting for the management of Long COVID syndrome, and that the use of simple and easily understood tools available to administer to patients is of fundamental importance.

This approach, if validated, could represent an alternative or a complementary model to hospital care (of which timing for treatment is delayed and less comfortable both in interpersonal and logistical aspects) and also more sustainable for the entire health system.

The findings reported in this contribution are very preliminary, emerging from a retrospective analysis performed by a group of General Practitioners in a territorial setting. The lack of a control group is the main limitation of this analysis; in fact it cannot be excluded that the symptom improvement was due to the natural recovery from Long COVID over time. Unfortunately, to our knowledge, the available literature data are various and often conflicting about the duration of Long COVID symptomatology, and the timing of its spontaneous resolution: on the one hand it is accepted that the symptoms can regress spontaneously; on the other it is evident that they can persist in important percentages [67]. However, PEALUT supplementation, by reducing the neuroinflammation underlying the pathology, might also have helped in improving patients symptoms, reducing the risk of the chronicization of COVID-19 sequelae.

## 5. Conclusions

Given its excellent tolerability profile and the lack of pharmacological interactions together with the numerous and previous evidence (preclinical and clinical) demonstrating the efficacy of PEA in the mitigation of non-resolving neuroinflammation, PEALUT might represent a valid tool for the General Practitioners in their daily clinical practice to support the recovery of patients complaining of Long COVID symptomatology.

The current clinical findings strongly support previous preclinical studies and emphasize the importance of work to be conducted on these compounds for the management of COVID-related complications.

Future randomized, prospective, controlled clinical trials are needed to confirm these preliminary results and verify the role of PEALUT in patients’ recovery from symptomatology and in the mitigation of the serious biopsychosocial impacts related to Long COVID Syndrome.

Furthermore, despite their limitations, the reported data provide the scientific community with the opportunity to reflect on some aspects which, for different reasons, simultaneously affect a person suffering from Long COVID, which can be summarized briefly below:

- the pathogenetic role of “non-resolving” neuroinflammation in Long COVID;
- the importance of neuroinflammation modulation through a substance such as umPEA;
- the primary role of the General Practitioner and the real-life setting in the early diagnosis and treatment of this pathology;
- the advantage of recognizing this key figure and the territorial setting as the most appropriate for the management of Long COVID syndrome.

## Figures and Tables

**Table 1:** Patients basal characteristics.

No. of Patients	Sex	Age (Mean ± S.D. *)
49 outpatients	18 males; 31 females	53.4 ± 12.0

S.D. * = standard deviation.

**Table 2:** Patient pre-existing pathologies.

Pre-Existing Pathologies	No. of Patients
Hypertension	10
Obesity	3
Hyperlipidemia	8
Diabetes Mellitus	5
Hypothyroidism	6
Anxiety	4
Depression	4
Chronic obstructive pulmonary disease	3
Osteoartritis (Diffuse, other than chronic low back)	6
Osteoporosis	7
Chronic Low Back Pain	6
Gastroesophageal reflux disease	4
Diverticulosis	2
Ulcerative colitis	1
Mieloma	1
Glaucoma	1

**Table 3:** Patient distribution according to pain course pattern at each time-point.

Pain Course Pattern		T0 (N = 49)	T60 (N = 46)	T90 (N = 42)
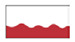	“Persistent pain with slight fluctuations”	11 (22.4)	13 (28.3)	9 (21.4)
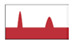	“Persistent pain with pain attacks”	8 (16.3)	3 (6.5)	2 (4.8)
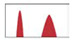	“Pain attacks without pain between them”	17 (34.7)	11 (23.9)	13 (31.0)
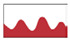	“Pain attacks with pain between them”	9 (18.4)	5 (10.9)	4 (9.5)
No pain		4 (8.2)	14 (30.4)	14 (33.3)

Value are expressed as N (%).

**Table 4:** Symptoms distribution among patients over time.

Symptoms Distribution	T0 (N = 47)	T60 (N = 46)	T90 (N = 45)	*p*
Fatigue	43 (91.5)	16 (34.8)	10 (22.2)	<0.0001
Weakness	35 (74.5)	17 (37.0)	7 (15.6)	<0.0001
Brain fog	30 (63.8)	8 (17.4)	5 (11.1)	<0.0001
Irritability	30 (63.8)	14 (30.4)	7 (15.6)	<0.0001
Difficulty multitasking	28 (59.6)	13 (28.3)	5 (11.1)	<0.0031
Memory loss	24 (51.1)	14 (30.4)	5 (11.1)	<0.0002
Anosmia	22 (46.8)	2 (4.3)	0	<0.0001
Dysgeusia	20 (42.6)	2 (4.3)	1 (2.2)	<0.0001
Inability to find the right word	20 (42.6)	14 (30.4)	5 (11.1)	<0.0025

Values are expressed as N (%), in order from the most to the least frequent at baseline (T0). T0: examination prior to start treatment with PEALUT; T60: examination after 60 days of treatment with PEALUT; T90: examination after 90 days of treatment with PEALUT.

## Data Availability

Data are available under request to the corresponding author.
